# Highly Concentrated Stabilized Hybrid Complexes of Hyaluronic Acid: Rheological and Biological Assessment of Compatibility with Adipose Tissue and Derived Stromal Cells towards Regenerative Medicine

**DOI:** 10.3390/ijms25042019

**Published:** 2024-02-07

**Authors:** Valentina Vassallo, Celeste Di Meo, Nicola Alessio, Annalisa La Gatta, Giuseppe Andrea Ferraro, Giovanni Francesco Nicoletti, Chiara Schiraldi

**Affiliations:** 1 Department of Experimental Medicine, Section of Biotechnology, University of Campania “Luigi Vanvitelli”, 80138 Naples, Italy; valentina.vassallo@unicampania.it (V.V.); celeste.dimeo@unicampania.it (C.D.M.); nicola.alessio@unicampania.it (N.A.); annalisa.lagatta@unicampania.it (A.L.G.); 2 Plastic Surgery Unit, Multidisciplinary Department of Medical, Surgical and Dental Sciences, University of Campania “Luigi Vanvitelli”, 80138 Naples, Italy; giuseppe.ferraro@unicampania.it (G.A.F.); giovannifrancesco.nicoletti@unicampania.it (G.F.N.)

**Keywords:** human adipose-derived stromal cells, differentiation process, tissue regeneration, low- and high-molecular-weight hyaluronic acid, highly concentrated hybrid complex, hyaluronidase-catalyzed degradation

## Abstract

Cells and extracts derived from adipose tissue are gaining increasing attention not only in plastic surgery and for aesthetic purposes but also in regenerative medicine. The ability of hyaluronan (HA) to support human adipose stromal cell (hASC) viability and differentiation has been investigated. However, the compatibility of adipose tissue with HA-based formulation in terms of biophysical and rheological properties has not been fully addressed, although it is a key feature for tissue integration and in vivo performance. In this study, the biophysical and biochemical properties of highly concentrated (45 mg/mL) high/low-molecular-weight HA hybrid cooperative complex were assessed with a further focus on the potential application in adipose tissue augmentation/regeneration. Specifically, HA hybrid complex rheological behavior was observed in combination with different adipose tissue ratios, and hyaluronidase-catalyzed degradation was compared to that of a high-molecular-weight HA (HHA). Moreover, the HA hybrid complex’s ability to induce in vitro hASCs differentiation towards adipose phenotype was evaluated in comparison to HHA, performing Oil Red O staining and analyzing gene/protein expression of PPAR-γ, adiponectin, and leptin. Both treatments supported hASCs differentiation, with the HA hybrid complex showing better results. These outcomes may open new frontiers in regenerative medicine, supporting the injection of highly concentrated hybrid formulations in fat compartments, eventually enhancing residing staminal cell differentiation and improving cell/growth factor persistence towards tissue regeneration districts.

## 1. Introduction

The application of human adult stem cells in regenerative medicine is emerging as a novel approach. To avoid complex implantation methods, interest in exploring the potential of stimulating the in vivo renewal of these stem cells by leveraging their existence within tissue-specific niches has been growing. This topic has garnered significant attention and prompted investigation within the scientific community. In fact, stem cells can be readily obtained from various adult tissues, including adipose tissue, the dermis, bone marrow, blood, and skeletal muscle [1]. This accessibility presents a potential solution to the challenges, regulations, and ethical problems associated with the use of embryonic stem cells (ESC) or induced pluripotent stem cells (iPSC) [2]. It is well-known nowadays that mesenchymal stem cells (MSCs) have the ability to differentiate between multiple cell types to enhance tissue regeneration [3]. Human adipose stromal cells (hASCs) are increasingly recognized for their efficacy in this setting due to their widespread presence throughout the human body and their capacity for larger-scale extraction in comparison to MSCs obtained from bone marrow [1]. Moreover, hASCs can be classified as a subset of MSCs and possess numerous regenerative capabilities [4]. These cells can be obtained through minimally invasive procedures and can be readily re-implanted and/or cultured in a laboratory-specific setting in order to increase the viable cell number after the in vitro transplant. For these reasons, hASCs have the potential to significantly contribute to the field of regenerative medicine due to several compelling factors. For instance, these cells have already been employed in autologous fat grafting procedures for aesthetic purposes, such as breast remodeling and addressing minor skin defects, as well as in reconstructive surgeries, including breast-conserving surgery and post-breast surgery, often resulting from cancer-related treatments [5,6,7]. Autologous fat injection, performed in a surgical setting, can be regarded as a procedure associated with minimal risk of immunological and rejection reactions [8]. The applications of hyaluronic acid (HA), a natural glycosaminoglycan (GAG) that plays a crucial role in our organism as both a structural component (e.g., in the dermal and cartilage extracellular matrix) and biological mediator (e.g., in tissue regeneration, as an anti-inflammatory agent, and in the commitment of stem cells to a specific phenotype), are continuously expanding also in the field of adipose tissue regeneration [9,10,11,12]. Hyaluronic acid can be employed in its linear configurations or subjected to chemical modifications to enhance its resistance to degradation and mechanical properties. Moreover, a combination of two distinct HA molecular weights, thermally stabilized in order to obtain hybrid cooperative complexes, also known as HCC, has also been recently obtained according to patented protocol [13]. Specifically, high-molecular-weight HA (e.g., 1400 kDa) and low-molecular-weight HA (e.g., 100 kDa) are mixed prior to undergoing thermal treatments that have been shown to optimize the hydrogen bonding cooperativity upon warming and cooling. In recent years, there has been a significant emphasis within the scientific community on the use of hyaluronic-acid-based hydrogels and scaffolds in the field of tissue engineering [14]. Furthermore, the application of three-dimensional (3D) cell culture methodologies has demonstrated successful preservation of pluripotency and/or differentiation markers in adult stem cells [15,16]. Within this framework, it has been demonstrated via recent investigations that hydrogels and scaffolds composed of HA have the ability to support the differentiation of adult stem cells towards a particular phenotype. This evidence is substantiated by the study conducted by Alessio et al. [9]. However, a prevalent concern associated with the in vitro culture of adult stem cells is the occurrence of senescence and a gradual decrease in their ability to differentiate into multiple cell types. This decline is primarily attributed to the prolonged expansion of these cells outside of their natural environment. Consequently, there is a growing demand for a gel or biomaterial that is both biocompatible and injectable directly into the tissue while also preserving its biophysical properties [17]. In the context of adipose regeneration, it is crucial to emphasize the requirement for a suitably viscous or rigid support or scaffold to ensure the retention of injected or implanted cells in a defined location or to facilitate prolonged interaction with resident stem cells, thereby enabling the completion of their intended functions. Regarding this matter, hydroxyapatite-based gels seem to be highly suitable for this purpose due to their widely recognized viscoelastic qualities, but they may induce inflammation through the NLRP3 inflammasome in vitro and in vivo [18]. However, compelling evidence indicates that HA, a key component of these products, does not elicit an immune response and undergoes total in vivo degradation within a span of 6 months [19]. Furthermore, previous studies have provided evidence indicating that HA possesses the capability to sustain an optimal milieu conducive to the in vitro development of hASCs [20,21]. This study aims to assess the biophysical, biochemical, and biological characteristics of Profhilo Structura^®^ (here briefly indicated as HL 90), a hybrid cooperative complex consisting of double-molecular-weight hyaluronic acid with a very high concentration of 45 mg/mL [22]. The evaluation was based on scientific protocols and approaches previously developed and published in the recent scientific literature [20,21]. A clinical study was recently published on HL 90 as a retrospective case series, assessing the bioremodeling performance of these intradermal formulations when injected in the facial fat compartment of healthy volunteers [23]. However, the present research report is the first in which the rheological behavior of adipose tissue was evaluated in combination with hyaluronic acid formulations. These measurements aimed to assess the capacity to maintain a consistent rheological profile while the hyaluronan complex was mixed to varying quantities of human adipose tissue, with the objective of ensuring compatibility with the tissue and also its potential use for in situ injection. Even more importantly, we assessed the capacity of stabilized HCC at high concentration to sustain the viability of hASCs and promote their differentiation towards an adipose phenotype. We compared this ability to that of high-molecular-weight hyaluronic acid (HHA) alone throughout a 21-day period of in vitro culture depicting the critical biomarkers array during differentiation.

## 2. Results

### 2.1. Rheological Characterization

**Figure 1:**
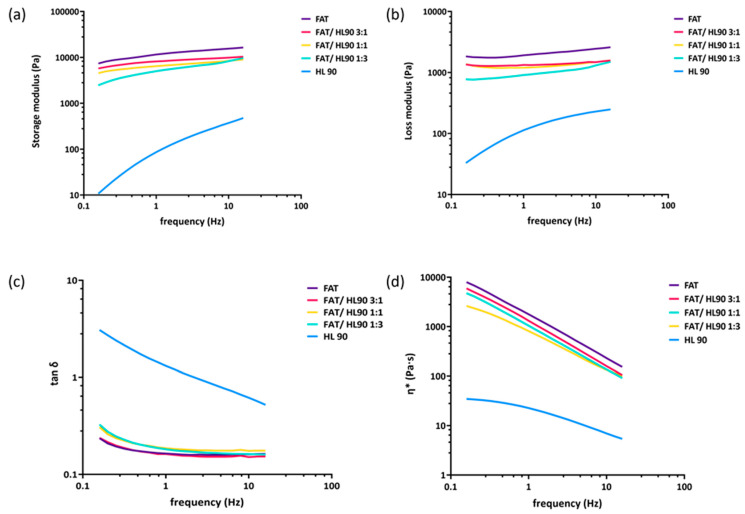
Rheological characterization of samples after a frequency sweep test at 37 °C. Elastic modulus (**a**); viscous modulus (**b**); complex viscosity (**c**); and tan δ as a function of frequency (0.159–15.9 Hz) at 1% of strain (**d**).

The results of the rheological measurements are shown in Figure 1. The storage modulus (Figure 1a), the loss modulus (Figure 1b), the complex viscosity (Figure 1c), and the tan δ (Figure 1d) of all the combinations of HL 90 with adipose tissue investigated were reported as a function of frequency in the range 0.159–15.9 Hz. The curves presented here were obtained by averaging all the rheological data achieved for each sample with at least three measurements.

Among the formulations, only the hyaluronan-based hybrid cooperative complex showed a viscoelastic behavior, with G″ that exceeded G′ at low frequencies and G′ that surpassed G″ at high values of frequency (up to 2.5 Hz, which corresponds to the crosso-ver point). The fat tissue presented the typical elastic features with a storage modulus that remained higher than the loss modulus in the whole range of frequency examined. The addition of HL 90 to the adipose tissue in the ratios tested did not affect the general rheo-logical behavior of the resulting preparations, which always resembled that of the fat tis-sue. The elastic modulus proved highly superior to the hyaluronan-based formulation alone and all the different ratio mixtures. Despite the curve, trends were perfectly con-served upon addition of the stabilized hybrid complex at high concentration to the fat tis-sue; the average resultant values of both dynamic moduli were lower than those of the single fat tissue in the whole range of frequency tested as the hyaluronan solution amount increased in the final formulation.

To simplify the comparison, the values of G′, G″, and η* measured at 1 Hz were re-ported in Table 1 as mean ± SD.

As would be expected in the presence of human tissue, the rheological data of all the fat-containing samples showed high variability among measurements. The statistical analyses carried out on the viscoelastic moduli and the complex viscosity at 1 Hz showed that HL 90 was significantly (*p*-values lower than 0.05) different from the fat. None of the samples obtained by combining the formulation with the fat, including the one with only 25% of fat (Fat/HL 90 1:3), presented statistical differences from the pure adipose tissue. The tan δ value at 1 Hz was averagely 1.1 for HL 90, which is lower than for the HA hybrid complex at 32 mg/mL (e.g., Profhilo^®^). 

#### Sensitivity to Enzymatic Degradation Studies

**Figure 2:**
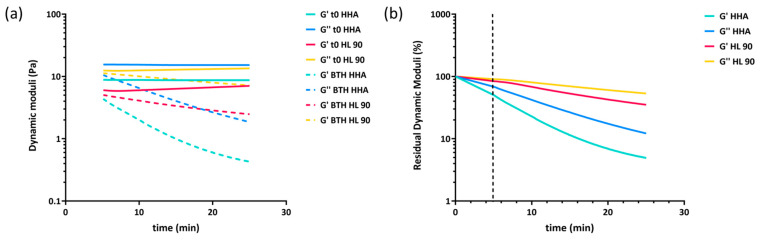
Reduction in storage and loss moduli during 25 min of incubation with BTH 10 U/mL for the formulations. G′ and G″ as a function of time recorded for the samples in the presence of the enzyme. Values measured during incubation with PBS under the same experimental conditions (control) were also reported. All measurements were carried out at 37 °C, 2.5 Hz of frequency, and 2% of strain. (**a**) Residual G′ and G″ as a function of time in 25 min incubation with BTH (**b**). The vertical dashed line separates the extrapolated values (on the left) from the data obtained by performing rheological measurements (on the right).

The extent of degradation of the HA hybrid complex (45 mg/mL) and a linear HHA-based solution due to enzymatic hydrolysis were studied by carrying out rheological measurements. Elastic and viscous moduli of the formulations during incubation with bovine testicular hyaluronidase (BTH) were monitored for 25 min to compare the stability of the samples. The control samples obtained by diluting the products 1:2 (*v*/*v*) with phosphate-buffered saline (PBS) in place of the BTH solution were also characterized. Figure 2 shows the results of the rheology-based comparison for all the samples under examination. Storage and loss moduli recorded for the controls remained constant throughout the time interval of observation for both samples, whereas, when incubated with the enzyme, HL 90 and the HHA-based solution reduced their dynamic parameters, exhibiting a similar degradation profile and indicating sensitivity to the BTH hydrolysis.

Although both samples demonstrated sensitivity to enzymatic activity, the rheological spectra highlighted a different extent of degradation between the samples; HL 90 proved more stable than HHA since the slopes of G′ and G″ profiles, as a function of time, were lower than those recorded during HHA degradation. The rate of G′ and G″ decrease for the enzymatic hydrolysis was also calculated and reported in Table 2 in terms of residual G′ and residual G″ percentages after 5, 15, and 25 min of treatment.

After 5 min of incubation, HL 90 maintained about 77% and 87% of its elastic and viscous moduli, while HHA retained only 50% and 68% of the initial values. Moreover, prolonged enzymatic hydrolysis (up to 25 min) showed that HL 90 still preserved around 38% G′ and 55% G″ in contrast with HHA-based solution, which showed values of G′ and G″ around 5% and 12% of the control, which showed them to be 5–7 times more depleted than the hybrid complex. These results demonstrated that the viscoelastic properties are better retained by the hybrid stabilized complex with respect to linear HA when subjected to hyaluronidase attack/enzymatic digestion.

**Figure 3:**
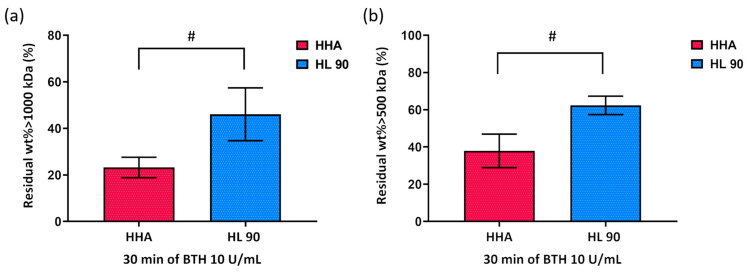
Weight fraction with molecular weight above 1000 kDa (**a**) and 5000 kDa (**b**) residue (%) after 30 min of incubation with BTH 10 U/mL. A *t*-test was used to compare the significance of the parameters of HL 90 with respect to HHA (# *p* < 0.05).

The results of the hydrodynamic characterization carried out on the samples after the enzymatic activity (30 min of incubation with BTH 10 U/mL) were reported in terms of residual fraction (wt%) of macromolecules with molecular weight above 500 kDa and 1000 kDa and shown in Figure 3. As obtained for the rheology-based comparison, both samples showed an important molecular weight degradation due to the enzymatic hydrolysis, with HL 90 demonstrating higher stability with respect to HHA. Specifically, the molecular fraction above 1000 kDa preserved for HHA was about 2-fold lower than HL 90 (23% vs. 46%, respectively), while the residual weight fractions higher than 500 kDa were found to be 38% for HHA and 62% for HL 90.

### 2.2. Adipogenic Differentiation

#### 2.2.1. Cellular Viability and Proliferation in Presence of HA-Based Gels

**Figure 4:**
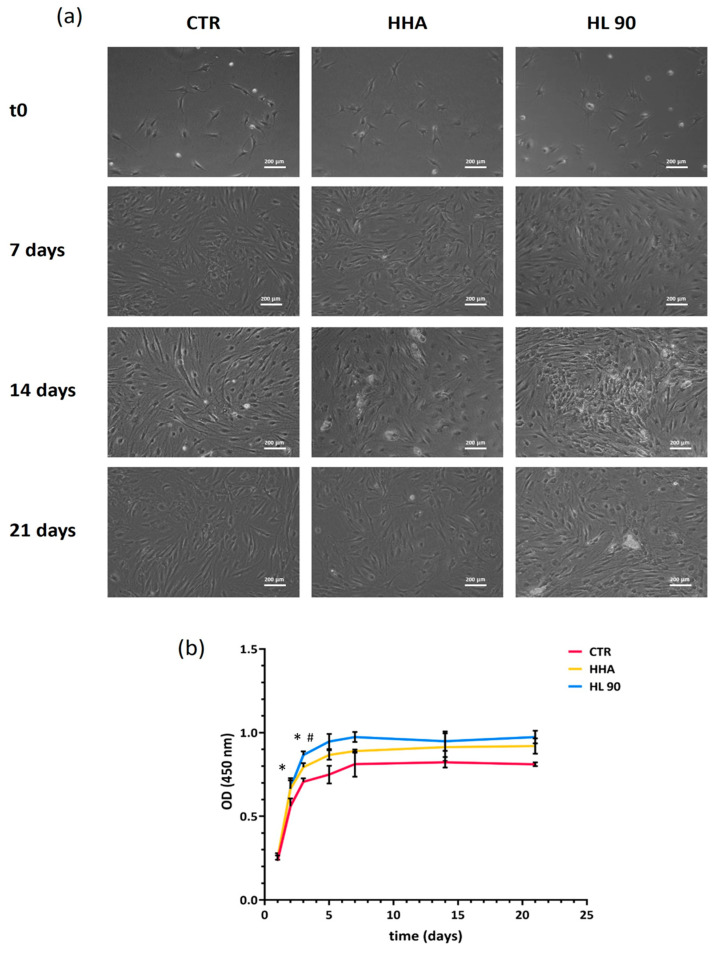
Cell viability assay performed using CCK-8 staining. Pictures of cells within the assay, scale bar 100 μm, 10× magnification (**a**). Cells’ growth curves (**b**). Results are presented as mean ± SD; *t*-test was used to compare the significance of each treatment with respect to CTR (* *p* < 0.05) and HL 90 vs. HHA (# *p* < 0.05).

As expected, being based on hyaluronic acid, both the formulations sustained hASCs viability consistent with previously published data [20], even at a higher initial concentration (22.5 mg/mL and 11.25 mg/mL vs. 5 mg/mL). Figure 4 shows that the cells grew until 14 days. As expected, the slowdown in cell growth, approaching cell confluence in the wells, was overlaying with the expression of key genes, such as PPAR-γ, which is generally known to be crucial to switch on stem cells’ differentiation into adipocytes. Specifically, the graph in Figure 4b displays that HL 90 significantly (*p* < 0.05) prompted the cells’ viability better than untreated cells (cultivated in the presence of culture medium diluted in a 1:1 ratio with PBS) already after 48 h and better than HHA-supplemented medium in prolonged incubation (72 h). Between 3 and 4 days of culture, the cell growth was significantly (*p* < 0.05) faster than the control, with the best performance for HL 90 (*p* < 0.05 vs. HHA). In addition, the hybrid complex was more efficient than HHA, with a cell viability increase vs. CTR of about 20% and 16% after 96 h and 7 days of incubation. Meanwhile, at same experimental points, HHA addition increased hASCs’ viability by about 11% and 7% with respect to CTR.

#### 2.2.2. Intracellular Lipid Accumulation Evaluation after HA-Based Gels Treatment (Oil Red O Staining)

**Figure 5:**
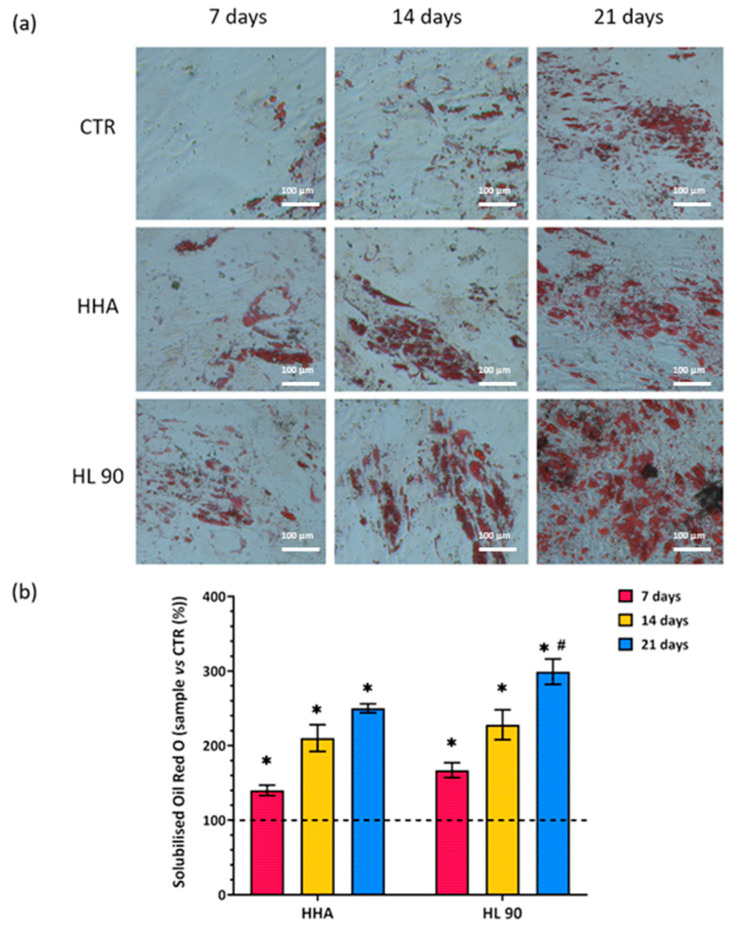
Oil Red O staining assay performed after 7, 14, and 21 days of in vitro culture. Pictures of cells within the assay, scale bar 100 μm, 20× magnification (**a**); solubilized Oil Red O (samples vs. CTR (%)) Dashed line present in the graph is related to untreated cells (CTR) (**b**). Results are presented as mean ± SD; *t*-test was used to compare the significance of each treatment with respect to CTR (* *p* < 0.05) and HL 90 vs. HHA (# *p* < 0.05).

Given that the information about cellular differentiation provided by the proliferation assay cannot be so exhaustive, Oil Red O staining was performed in order to evaluate the intracellular lipid accumulation. As shown by the pictures in Figure 5a, there is a progressive increase in intracellular lipid droplets within the incubation for both the treatments in comparison to untreated cells. Data related to Oil Red O solubilization, reported in Figure 5b, showed that the major amount of lipid droplets was found after 21 days of incubation for HL 90-treated samples. Specifically, this was significantly higher than CTR (*p* < 0.05) by about 3-fold. Moreover, at this experimental time point, the lipid droplets content of HL 90 significantly (*p* < 0.05) increased with respect to HHA treatments.

#### 2.2.3. Adipogenic Gene Expression Analyses by qRT-PCR

**Figure 6:**
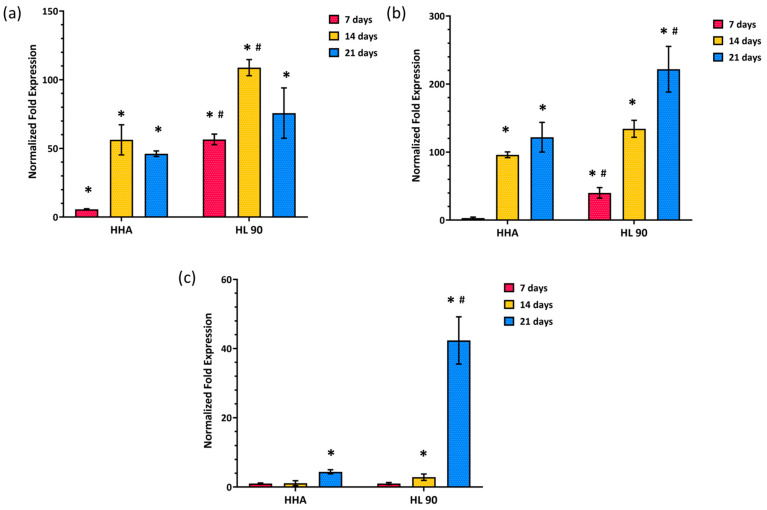
Expression analyses by qRT-PCR of genes having a key role in the differentiation process versus adipose phenotype PPAR-γ (**a**), ADP (**b**), and LP (**c**). Data are normalized with respect to untreated cells (CTR). Results are presented as mean ± SD; *t*-test was used to compare the significance of each treatment with respect to CTR (* *p* < 0.05) and HL 90 vs. HHA (# *p* < 0.05).

The quantitative real-time polymerase chain reaction (qRT-PCR) outcomes showed that the HA-based products were able to sustain the differentiation of hASCs versus adipose phenotype through the gene modulation of all the biomarkers selected in this study. Being an early adipogenic enhancer, the PPAR-γ gene expression was upregulated within 7 and 14 days, while it decreased after 21 days in the in vitro culture in the presence of the tested hydrogels. In detail, the PPAR-γ gene expression increased in comparison to CTR after 7 days. It seemed that the central/turning point of the differentiation process was reached at 14 days with an upregulation of about 56- and 108-fold vs. CTR in the presence of HHA and HL 90, respectively (Figure 6a). Both at 7 and 14 days of treatment, PPAR-γ in the presence of HL 90 was significantly (*p* < 0.05) higher than HHA. On the other hand, the modulation trend of adiponectin (ADP) and leptin (LP) was different with a progressive increase within that time. In fact, the ADP expression level prompted starting from 14 days with an increase of about 96-fold vs. CTR with HHA alone and 134-fold vs. CTR with the HA hybrid complex (45 mg/mL). Figure 6b shows a further increase in this biomarker, specifically of about 121- and 221-fold vs. CTR after 21 days of incubation with HHA and HL 90, respectively, with a significant difference (*p* < 0.05) between the samples. Finally, the LP gene expression was also shown to be most enhanced after 21 days in the in vitro culture in the presence of the linear HA (5-fold vs. CTR) and the HL 90 (*p* < 0.05) (42-fold vs. CTR) (Figure 6c). For this biomarker, the gene modulation effect in the presence of HL 90 was significantly different (*p* < 0.05) from HHA after 21 days. 

#### 2.2.4. Adipogenic Protein Evaluation

##### PPAR-γ, ADP, and LP Expression Evaluation by Western Blotting

**Figure 7:**
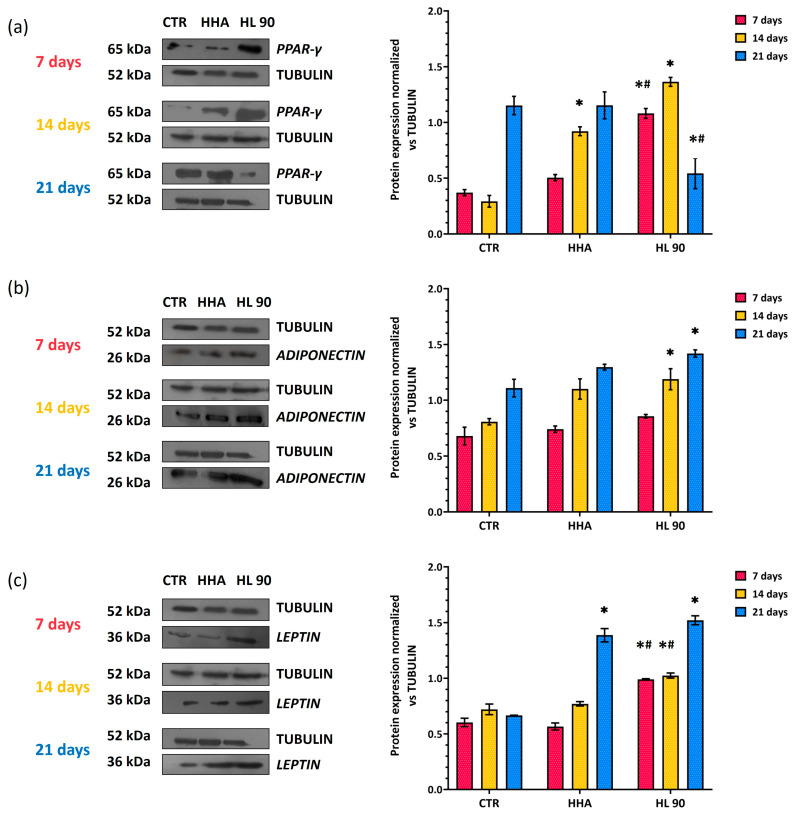
Expression levels evaluation by WB of protein related to the adipogenic differentiation; PPAR-γ (**a**), ADP (**b**), and LP (**c**) in hASCs grown in vitro for 7, 14, and 21 days in presence of HHA or HL 90. Densitometric analyses were performed normalizing each protein expression with respect to tubulin. Data are presented as mean ± SD. *t*-test was used to compare the significance of each treatment with respect to CTR (* *p* < 0.05) and HL 90 vs. HHA (# *p* < 0.05).

To better explore the modulation of specific analytes involved in the differentiation process versus the adipose phenotype, PPAR-γ, ADP, and LP expressions were also analyzed by Western blotting (WB). According to the qRT-PCR data, the PPAR-γ protein expression decreased within the time, while ADP and LP increased (Figure 7). In detail, the cells incubated in the presence of HL 90 showed a significant (*p* < 0.05) upregulation of PPAR-γ already after 7 days in comparison to CTR as well as the HHA sample; meanwhile, in the HHA-treated cells, its production level was shown to be very similar to untreated cells (Figure 7a). These data may prove that the HA hybrid complex (45 mg/mL) prompted the differentiation process better or faster in comparison to hyaluronan alone. On the contrary, after 21 days of in vitro culture, the HL 90-treated cells displayed a significant (*p* < 0.05) reduction in PPAR-γ expression in comparison to CTR and HHA; again, in the presence of HHA, the level of this biomarker was comparable to untreated cells, perhaps due to a delay in the activation of the differentiation pathway (Figure 7a). The ADP protein level was higher after 14 and 21 days in comparison to 7 days in the presence of both products (Figure 7b). Furthermore, ADP upregulation with respect to CTR increased about 1.37-fold and 1.50-fold (*p* < 0.05) with HHA and HL 90, respectively, after 14 days of stimulation. In addition, as shown in Figure 7b, after 21 days of in vitro culture, the ADP protein expression continued to be increased, specifically by about 1.22-fold and 1.34-fold (*p* < 0.05) vs. CTR in the presence of HHA and HL 90, respectively. Finally, both the tested samples induced a significant (*p* < 0.05) increase in the LP protein level after 21 days of stimulation; in detail, an increase of 2.01- and 2.26-fold in the presence of the HA alone and HL 90, respectively (Figure 7c), was observed. The LP protein increase after 14 and 21 days of stimulation was significantly (*p* < 0.05) marked in HL 90-treated cells in comparison to HHA ones (Figure 7c).

##### ADP and LP Secretion Evaluation by ELISA Assay

**Figure 8:**
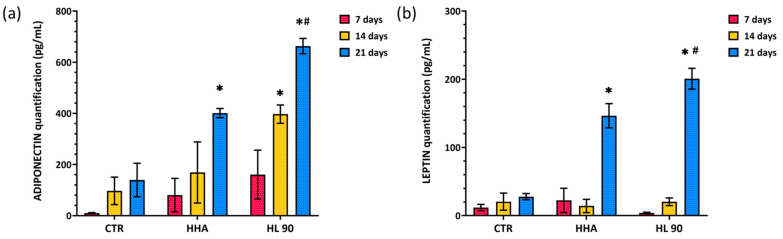
ELISA assay relative to ADP (**a**) and LP (**b**) after 7, 14, and 21 days of in vitro culture. Data are reported as mean ± SD; *t*-test was used to compare the significance of each treatment with respect to CTR (* *p* < 0.05) and HL 90 vs. HHA (# *p* < 0.05).

As well as the WB results, ELISA assays confirmed an increasing secretion of ADP and LP at prolonged incubation time (Figure 8). In detail, both the HA-based formulations induced a strong secretion of ADP in comparison to CTR at 21 days. Specifically, HHA treatment resulted in about a 2.87-fold increase when compared to CTR, while HL 90 prompted 4.57-fold ADP secretion; thus, both were significantly higher (*p* < 0.05) than CTR (Figure 8a). Also, for LP protein secretion, after 21 days of in vitro culture, HHA and HL 90 treatments proved 5.6-fold and 7.2-fold higher than untreated cells, respectively. In this case, the LP amount was significantly greater for HL 90-treated cells than HHA-treated ones (*p* < 0.05) (Figure 8b).

## 3. Discussion

In recent years, the field of adipose tissue engineering has emerged as a promising approach to facilitating tissue regeneration and reconstruction. This may involve direct stimulation of the resident stem cell niches in the fat compartments (e.g., facial ones) or the extraction and in vitro expansion of hASCs, which are subsequently reintroduced into a targeted region of the body with the aid of a hydrogel or scaffold [24,25,26]. 

The biophysical characteristics of a prospective cellular support/scaffold are contingent upon both its intended function and the specific location of implantation [27]. The evaluation of viscoelastic behavior is considered a crucial feature. The concept is tightly linked to the physiological reaction to stress, which is associated with the specific body or tissue location and impacts the ease of administering injections using thin needles [28]. As previously elucidated, adult stem and stromal cells cultivated in vitro necessitate a sophisticated matrix to ensure adequate oxygen and nutrient exchange, intercellular communication, and, ultimately, differentiation into a distinct phenotype. In this context, the use of HA proved highly advantageous due to its inherent biocompatibility, biodegradability, and amenability to chemical modifications [13,29,30,31]. Regrettably, in some instances, cells cultivated in vitro for an extended period experience senescence and/or a loss of their multipotency [19]. Therefore, an ideal candidate would be a support material that possesses distinct viscoelastic properties, exhibits long-term persistence in vivo, can accommodate cells (whether they are in vivo resident or ex vivo implanted), and simultaneously promotes their differentiation towards a desired phenotype. Taking into consideration the extensively studied in vitro biological impacts of HCCs, such as their ability to maintain skin elasticity, enhance wound healing, protect muscle cells from stressful agents, and induce adipogenic differentiation of hASCs, this study focuses on the comprehensive characterization of a novel highly concentrated hybrid cooperative complex formulation. It is composed of unmodified high- and low-molecular-weight HA; however, such a highly concentrated formulation has never been reported in the literature previously, and, therefore, it was fully characterized in the present work, with a specific interest in investigating its capacity to induce hASCs differentiation towards the adipose phenotype as compared to linear hyaluronan of high molecular weight. The biomechanical characterization has the potential to aid in the prediction of the formulation’s behavior during and following in vivo administration, thereby playing a crucial role in determining its therapeutic efficacy [32,33]. Indeed, G′ represents the product stiffness, which refers to its ability to withstand elastic deformations during the injection process and its capacity to uphold the physical structure in response to tissue movements following implantation. The parameter η* has a strong correlation with the hydrogel capacity to withstand shear forces resulting from the needle during the implantation process, as well as the physiological forces that occur in vivo after the injection [33,34]. The biophysical characteristics and spatial distribution of adipose tissue exhibit considerable variability, contingent upon factors such as the site of extraction, the individual’s age, gender, and ethnicity, their overall health condition, and the specific technique employed for extraction [35]. Under the experimental conditions employed, the adipose tissues analyzed exhibited the typical characteristics of an elastic solid. Specifically, they demonstrated the ability to store energy elastically and effectively restore the original shape following deformation induced by external forces upon their removal. On the other hand, it appears that, at a high concentration (45 mg/mL), the HA hybrid complex showed the properties of a viscous liquid as expected, but the entanglement seems to be superior to that of HCC (32 mg/mL), thus displaying a potentially better mechanical performance and reducing deformation under the influence of external forces. As a matter of fact, HL 90 shows higher G′ at 1 Hz and lower tan δ than HCC (32 mg/mL); in addition, its curve of tan δ versus frequency (>2.5 Hz) showed lower values than the one recorded for Sinovial HL^®^ [18,21]. These data suggest a further little step, with this higher concentration of the hybrid complexes, towards the rheological characteristics of low crosslinked soluble gels. It is interesting to note that HL 90 alone showed lower moduli than the human adipose tissue. However, upon its mixing with human-derived fat, even in different proportions, the observed rheological characteristics resembled the ones of the original tissue without any significant difference. This finding holds substantial therapeutic implications as it makes the product desirably usable for the intended uses (e.g., facial fat pad replenishment and beyond breast regenerative medicine).

In addition, it was observed that the HA hybrid complex (45 mg/mL) demonstrated greater stability when exposed to hyaluronidase compared to solutions based on linear high-molecular-weight hyaluronan. Despite being susceptible to enzymatic breakdown, thus ensuring reabsorption, HL 90 exhibited a slower hydrolysis rate, keeping higher molecular weight HA fractions, thus maintaining the viscoelastic properties longer. The direct correlation between increased resistance to hyaluronidase hydrolysis, which is a key enzyme involved in HA degradation and extended in vivo persistence, has also been established in a murine model [22]. Moreover, when the formulations exhibit the capability to ensure prolonged biomechanical performance, the duration of the treatment outcome may be increased, thereby improving the efficacy of the clinical intervention, and reducing the frequency of injections in a therapeutic regimen. The process of differentiation into the adipogenic phenotype is an intricate and multifaceted phenomenon that encompasses various genes and proteins in specific biochemical pathways. In our experimental configuration, it is noteworthy that the modulation of biomarkers indicated that the presence of hyaluronan facilitated the in vitro differentiation of hASCs within a period of 21 days, even when used at high concentration. According to Siersbaek [36] and Rosen [37], PPAR-γ is recognized as an early factor in the adipogenesis process, whereas ADP and LP are generated at a later stage [38]. In the present study, we observed that HL 90 exhibited a greater capacity for enhancing differentiation by upregulating PPAR-γ expression after 14 days and subsequently achieving the maximum expression levels of ADP and LP after 21 days of incubation, when the early biomarkers were definitely downregulated. Furthermore, Western blotting and enzyme-linked immunosorbent assay (ELISA) techniques were employed to confirm that the formulations under investigation exhibited enhanced protein production and secretion associated with the adipogenic process. Notably, the HA hybrid complex at high concentration showed superior effectiveness in this regard. The results were validated through the utilization of Oil Red O staining, a technique that effectively visualizes the augmented intracellular buildup of lipid droplets in samples treated with the hybrid complex at elevated concentration. The results supported the potential efficacy of this concentrated formulation in promoting adipogenesis. This may imply that the injection of a high quantity of complexed hyaluronan is not detrimental to human cells, including the more delicate stromal cells. It did not seem to hamper proliferation and differentiation despite the high-viscosity environment. Indeed, the viability of hASCs increased, and the biomarkers associated with adipogenesis confirmed our hypothesis. The overall framework, coming from the in vitro experiments conducted here, paved the way for the application of hyaluronan and especially HL 90 in targeted areas of the face or body to address either tissue augmentation and/or replenish small defects, generally supporting rejuvenation/regeneration of fat compartments depleted in the aging process. In fact, this formulation showed promise in terms of improving tissue integration, extended stability, and enhanced differentiation of the stromal cells present in adipose tissue. These findings support the preliminary clinical experience with HL 90, which demonstrated optimal spreading and integration into the interstitial spaces of the fat compartment, stimulating an increase in its thickness [38]. An additional hypothesis suggests that combining HA hybrid complex (45 mg/mL) with hASCs and/or adipose cells obtained in the surgical setup from the patient for reinjection may enhance the viability and functionality of the transplanted adipose tissue/cells, thus better supporting tissue remodeling and regeneration procedures. However, to comprehensively address this particular use, it is imperative to acquire robust clinical evidence. The in vitro evidence presented in this study emphasized that the biophysical characteristics observed for the highly concentrated hybrid complex are slightly diverse when compared to linear HHA and also to the HCC previously characterized at lower concentrations. This suggests that injecting HL 90 deeper provides a better “pillar” for tissues, even if not crosslinked and thus soft and spreadable. Moreover, the biochemical and biological sound features support advantageous impacts of the tested formulation in highly responsive cell types derived from humans.

## 4. Materials and Methods

### 4.1. HA-Based Materials

The formulations tested here are composed of highly purified hyaluronic acid.

Specifically, the first product, Profhilo Structura^®^ (briefly HL 90), was provided by IBSA Farmaceutici Italia (Lodi, Italy) in sterile syringes. It is a hybrid cooperative complex (45 mg/mL final concentration) obtained by a patented NaHyCo^®^ Technology based on thermal treatment using as starting points a high-molecular-weight (1200 ± 100 kDa) and a low-molecular-weight (100 ± 10 kDa) hyaluronic acid [12].

The second sample is a pharmaceutical-grade high-molecular-weight linear hyaluronan (HHA) of 1200 ± 100 kDa, obtained by dissolving a specific amount of powder (SHYALT^®^ Altergon Italia Srl, Avellino, Italy) in PBS (22.5 mg/mL final concentration) and sterilizing the resulted solution for 12 min at 121 °C in autoclave (Vapormatic 770). 

### 4.2. Materials

All the materials used in this study were provided by Gibco (Carlsbad, CA, USA) unless otherwise stated.

Bovine testicular hyaluronidase salt-free lyophilized powder was purchased from Sigma-Aldrich, Milan, Italy.

### 4.3. Adipose Tissue

Human subcutaneous adipose tissues, generated as post-plastic surgery waste and generally obtained from breast, were kindly provided by the Plastic and Reconstructive Surgery Clinic of University of Campania “Luigi Vanvitelli” (Naples, Italy). The tissues were collected by lipectomy (from two female patients 45 and 55 years old, with a mean BMI of about 25 kg/m^2^), and all the procedures were approved by the Internal Ethical Committee of the University of Campania (formerly called Second University of Naples). Internal Registry: Experimentation #914. All the procedures were in line with the principles of the Declaration of Helsinki. 

### 4.4. Rheological Characterization 

#### 4.4.1. Sample Preparation

The HA hybrid complex (45 mg/mL) and the fat tissue were analyzed for a rheological characterization as they were received and in combination at different ratios, as follows: (a) HL 90: fat tissue 3:1 (*w*/*w*); (b) HL 90: fat tissue 1:1 (*w*/*w*); (c) HL 90: fat tissue 1:3 (*w*/*w*).

Specific amounts of the two components were placed in a 5 mL Eppendorf and mixed by stirring at 37 °C and 600 rpm until the fat absorbed the hyaluronan solution completely.

#### 4.4.2. Rheological Measurements

The mechanical spectra for all the formulations investigated were derived using an MCR 301 Physica oscillatory rheometer from Anton Paar (Ostfildern-Scharnhausen, Germany). A 25 mm parallel-plate geometry with a 1.0 mm gap was used, and the temperature was controlled via a Peltier system and kept at 37 °C. The rheometer was monitored during the measurements, and the data were collected with the Anton Paar software “RheoCompass™” (version 1.30.1227-Release). Oscillation tests were obtained over a frequency range of 1 to 100 rad/s (0.159 to 15.9 Hz) at 1% of strain, and the profiles of the storage modulus, the loss modulus, the tan δ, and the complex viscosity were determined as a function of frequency. The values of these parameters at 1 Hz were also extracted from each measurement and reported as mean ± SD. Samples were injected using a 21-gauge needle, and at least 3 separate preparations of each formulation were measured for checking the reproducibility and compatibility of the results.

#### 4.4.3. Stability to Enzymatic Degradation

The sensitivity of HL 90 to BTH degradation was tested through rheological measurements, following the protocol reported by La Gatta et al. [18]. Specifically, sample rheological parameters (elastic and viscous moduli) were monitored during the time in the presence of the enzyme with a final concentration of 10 U/mL. A BTH solution in PBS (Dulbecco phosphate buffer solution without calcium and magnesium) was previously prepared at 20 U/mL and added to the sample in gel dilution ratio of 1:2 (*v*/*v*); the formulation was gently stirred for 2 min before placing it in the rheometer at 37 °C. The measurements were carried out after exactly 5 min from the BTH addition. Storage and loss moduli were derived as a function of time at a constant frequency of 2.5 Hz, a constant strain of 2%, and 37 °C. A cone–plate geometry (diameter 49.970 mm, angle 1.995°, and truncation 207 μm) was used for the measurements. Hybrid cooperative complex 45 mg/mL was analyzed at least in triplicate to assess reproducibility, and a solution of linear HHA at 22.5 mg/mL (the same concentration of the high-molecular-weight hyaluronan in the complex) was also studied for comparison. The same test was performed for each sample by diluting the solution in PBS (1:2 *v*/*v*) without any addition of BTH to achieve the rheological behavior of the control. Sample degradation was evaluated by following the rheological parameters’ decrease during time with respect to the corresponding control. The residual values of G′ and G″ after 5, 15, and 25 min from the incubation with the enzyme were calculated as follows:

(1) $$Residual\;G^{\prime }\left(t\right)\left(\%\right)=\frac{{G^{\prime }}_{BTH}(t)}{G^{\prime }(t=0)}\times 100$$

(2) $$Residual\;G\prime \prime \left(t\right)\left(\%\right)=\frac{{G\prime \prime }_{BTH}(t)}{G\prime \prime (t=0)}\times 100$$

where G′(t) and G″(t) indicate the storage and loss moduli at a certain incubation time, and G′(t = 0) and G″(t = 0) are the values of the viscoelastic moduli obtained for the control samples in the same experimental conditions. 

Moreover, all the samples were analyzed for a detailed hydrodynamic characterization using a size exclusion chromatography triple detector array (SEC-TDA, Viscotek, Malvern, PA, USA). Specifically, after 30 min of treatment, HL 90 and HHA solutions in the presence of BTH 10 U/mL and PBS (control samples) were withdrawn and boiled for 10 min to stop the enzyme hydrolytic activity, filtered on 0.22 μm membranes, and analyzed by SEC-TDA. The extent of degradation was evaluated as the decrease in the fraction (wt%) of macromolecules with molecular weight above 500 kDa and 1000 kDa between control and treated samples. The residual percentage was determined as follows:

(3) $$Residual\;{wt\%\;}_{Mw>500\;kDa}\left(\%\right)=\frac{{wt\%\;}_{Mw>500\;kDa}(post\;BTH)}{{wt\%\;}_{Mw>500\;kDa}(control)}\times 100$$

(4) $$Residual\;{wt\%\;}_{Mw>1000\;\;kDa}\left(\%\right)=\frac{{wt\%\;}_{Mw>1000\;kDa}(post\;BTH)}{{wt\%\;}_{Mw>1000\;kDa}(control)}\times 100$$

where wt% _Mw>500 kDa_ (post-BTH) and wt% _Mw>1000 kDa_ (post-BTH) indicate the fraction (wt%) of macromolecules with molecular weight above 500 kDa and 1000 kDa, respectively, after 30 min of incubation with BTH 10 U/mL, while wt% _Mw>500 kDa_ (control) and wt% _Mw>1000 kDa_ (control) represent the same molecular fraction of the samples diluted in PBS.

Calculations were conducted on each measurement replicate, and results were reported as mean ± SD. The statistical analysis was carried out using a *t*-test.

### 4.5. Mesenchymal Stromal Cells Isolation from Human Adipose Tissue

The primary cells were obtained following the experimental protocols previously described by Stellavato et al. (2017) with slight modification [20]. In detail, fat tissues were washed with PBS, scraped, and enzymatically digested by a solution composed of collagenase type I at 3 mg/mL and dispase at 4 mg/mL, at 37 °C temperature, on a shaking plate for one hour. Then, the cell suspension was filtered (70 μm, BD, Falcon, Franklin Lakes, NJ, USA), centrifuged at 1500 rpm for 10 min (Eppendorf, Hamburg, Germany), washed with PBS, and re-centrifuged. The obtained cell pellet was re-suspended in Dulbecco’s modified eagle medium (DMEM) low glucose, supplemented with Fetal Bovine Serum (FBS) (10% *v*/*v*), penicillin–streptomycin (1% *v*/*v*), and Amphotericin B (1% *v*/*v*) in a 25 cm^2^ standard flask.

#### Cell Characterization

The specific phenotype of human adipocyte stromal cells (hASCs) was confirmed by flow cytometry as reported by Alessio et al. 2018 [9]. Briefly, the cells were washed with PBS and incubated with anti-CD105, anti-CD90, anti-CD73, anti-CD45, or anti-CD44 PE-conjugated antibody (Elabscience, Houston, TX, USA) according to the manufacturer’s instructions for 30 min in the dark at room temperature. Then, cells were re-washed with PBS and re-suspended in FACS buffer on a Guava easyCyte flow cytometer (Merck Millipore, Burlington, MA, USA) for data acquisition. The analysis was performed following a standard procedure using easyCyte software version 2.6. A minimum of 6000 cells for biomarker were analyzed and gated for forward-scatter versus side-scatter channel signals. The cells used for the experiment were at the third phase of in vitro culture. The results of the cell characterization are reported in Figure S1.

### 4.6. Adipogenic Differentiation

In order to assess the differentiation process towards the adipogenic phenotype, 50 × 10^3^ hASCs were gently mixed to 500 µL of linear HHA or HA hybrid complex (HL 90) through a sterile syringe. The samples containing the cells were seeded in a 6-well standard plate, and the adipogenic medium was added to dilute the HA-based formulations 1:2-fold (the final concentrations of HHA and HL 90 were 11.25 mg/mL and 22.5 mg/mL, respectively). The specific adipogenic culture medium was composed of DMEM medium (EuroClone, Naples, Italy) supplemented with HS (10% *v*/*v*) (Horse Serum) (EuroClone, Naples, Italy), penicillin/streptomycin (1% *v*/*v*) (EuroClone, Naples, Italy), 1 mM dexamethasone (Sigma-Aldrich, Saint Louis, MI, USA), 10 µg/mL insulin (Sigma-Aldrich, Saint Louis, MI, USA), 0.5 mM 3-isobutyl-1-methylxanthine (Sigma-Aldrich, Saint Louis, MI, USA), and 200 µM indomethacin (Sigma-Aldrich, Saint Louis, MI, USA). The treated cells (hASCs encapsulated in the hydrogels) and untreated cells (control CTR, hASCs grown in adipogenic culture medium diluted 1:2 with PBS, not encapsulated in the hydrogel) were cultured in vitro for 21 days, and the culture medium was changed every 3 days.

#### 4.6.1. Cellular Viability Assay

Cells’ viability was assessed within the differentiation process. In this respect, Cell Counting Kit-8 (Dojindo EU GmbH, Munchen, Germany) was used following the manufacturer’s protocol. Data were collected after 24, 48, 72, and 96 h and after 7, 14, and 21 days of in vitro culture. The optical densities of the obtained solutions were measured at 450 nm using a Beckman DU 640 spectrometer (Beckman, Milano, Italy). The relative cell viability was calculated as a percentage of the maximal absorbance:

(5) $$Viability=\frac{mean\;OD\;treated\;cells}{mean\;OD\;control}\times 100$$

Within the assay, the cells were observed by microscope (Leica DMi1, Wetzlar, Germany) at magnification 10× and relative pictures were captured by integrated camera (Leica MC120 HD). This assay was performed in duplicate and the results shown as mean ± SD of two different experiments. 

#### 4.6.2. Oil Red O Staining

The hASCs differentiation process versus the adipocyte phenotype was evaluated by Oil Red O staining (0.5% *v*/*v*) (Sigma Aldrich, Milan, Italy); in this way, lipid droplets present in cells were colored in red. Specifically, after 7, 14, and 21 days, the cells were washed three times with PBS in order to remove all the viscous solution and fixed with a solution of paraformaldehyde (4% *v*/*v*) in PBS for 20 min at room temperature. Oil Red O solution was incubated for 15 min at room temperature. After that, the stained lipid droplets were solubilized by using isopropanol (100% *v*/*v*) and quantified by measuring the absorbance at 510 nm through a Beckman DU 640 spectrometer (Beckman, Milan, Italy). The experiment was performed in duplicate and the results shown as mean ± SD. However, before the solubilization, in order to verify that red coloring occurred, the cells were observed by microscope (Leica DMi1, Wetzlar, Germany) at magnification 20× and relative pictures were captured by integrated camera (Leica MC120 HD). 

#### 4.6.3. Gene Expression Analyses by qRT-PCR

The analyses of specific genes expression were realized as previously reported [18]. In detail, after 7, 14, and 21 days of in vitro culture, 1000 μL of TRIzol^®^ reagent (Invitrogen, Milan, Italy) were added to each well, the controls, and the HA-based formulation encapsulating hASCs, followed by homogenization using a sterile pestle. After that, all the content of the well was transferred into sterile 1.5 mL tubes, 200 μL of chloroform were added, the tubes were gently shaken, and centrifuged at 12,000 rpm for 15 min at 4 °C (Eppendorf, Hamburg, Germany). Then, the supernatants containing mRNA were collected into clean 1.5 mL tubes diluted with 500 μL of isopropanol and left at −20 °C for 2 h. Successively, the samples were further centrifuged at 12,000 rpm for 1 h at 4 °C (Eppendorf, Hamburg, Germany), the supernatants were discharged, and the mRNA pellets were washed with EtOH 75% (*v*/*v*), centrifuged a 7500 for 5 min at 4 °C and left drying in ice under the chemical hood. Finally, mRNA pellets were re-suspended in nuclease-free purified water. The concentration of isolated RNA was evaluated by a Nanodrop Instrument (Celbio, Milan, Italy), and the cDNA was obtained using the Reverse Transcription System Kit (Promega, Milan, Italy) following the manufacturer’s instructions. Thus, a qRT PCR was performed using the IQ™ SYBR^®^ Green Supermix (Bio-Rad Laboratories, Milan, Italy). In this regard, each sample was analyzed in triplicate, and the mRNA expression of the following genes was normalized with respect to the glyceraldehyde-3-phosphate dehydrogenase (GAPDH) housekeeping gene: PPAR-γ, adiponectin (ADP), and leptin (LP). The specific primer sequences are reported in Table 3. The modulation of gene expression was evaluated through Livak’s method 2^−ΔΔCt^ (ΔΔCt = difference of ΔCt between treated cells and control) using Bio-Rad iQ5 software version 1.0 (Bio-Rad Laboratories, Milan, Italy) [39]. The experiment was performed in duplicate and the results shown as mean ± SD.

#### 4.6.4. Protein Expression Analyses

##### Western Blotting (WB)

Following the same experimental points of gene expression analyses, Western blotting (WB) was performed as previously described [18]. Thus, hASCs were harvested and lysed by a Radio-Immunoprecipitation Assay buffer (RIPA buffer 1×) (Cell Signaling Technology, Danvers, MA, USA). Protein concentration of each sample was determined through Bradford method [40]. For all samples, 30 µg of proteins were loaded on 12% SDS-PAGE gels, electroporated, and transferred to the nitrocellulose filters (GE, Amersham, UK). A solution based on 5% nonfat milk in Tris-buffered saline and 0.05% Tween-20 (TBST) was used to block the filters (1 h at room temperature), so these were incubated with antibodies against peroxisome-proliferator-activated receptor (PPAR-γ; Santa Cruz Biotechnology, Santa Cruz, CA, USA, 1: 250 *v*/*v*), adiponectin (ADP; Santa Cruz Biotechnology, Santa Cruz, CA, USA, 1:500 *v*/*v*), and leptin (LP, Santa Cruz Biotechnology, Santa Cruz, CA, USA, 1:250 *v*/*v*) overnight at 4 °C. After that, the membranes were washed with TTBS and specific horseradish peroxidase-conjugated anti-rabbit and anti-mouse antibodies (Santa Cruz Biotechnology, Dallas, TX, USA, 1:10,000 *v*/*v*) were incubated for 1 h at room temperature. Each blot was developed by the ECL system (Elabscience, Huston, TX, USA), and tubulin antibody (Santa Cruz Biotechnology, Dallas, TX, USA, diluted 1:1000) was used as gel loading control. Lastly, the semi-quantitative protein expression analysis was accomplished through Image J software version 1.8. This assay was performed in duplicate and the results shown as mean ± SD of two different experiments.

##### ELISA Assay

The ADP and LP levels in the cellular supernatants were measured using the ELISA assay. In this respect, after 7, 14, and 21 days of in vitro culture, the supernatants were recovered, centrifuged, and collected at −20 °C. ELISA Kits (Elabscience, Huston, TX, USA) were used to quantify ADP and LP according to the manufacturer’s instructions. The experiment was performed in duplicate and the results shown as mean ± SD.

### 4.7. Statistical Analyses

For the rheological measurements, the software Jasp 0.16.4.0 was used for the ANOVA test. Specifically, the values of dynamic moduli and complex viscosity at 1 Hz of frequency were compared for all the samples, and *p*-values less than 0.05 were considered significant. All the available corrections (Tukey, Bonferroni, Scheffé, Holm, and Šidák) were utilized.

For the biological evaluation, a *t*-test was performed to compare the results obtained for the hyaluronan-based treatments and the untreated cells, and between HL 90 and HHA.

*p*-values less than 0.05 were considered significant.

## 5. Conclusions

In vitro studies based on MSCs derived from human adipose tissue are relevant for deepening our understanding of the functional role of different hyaluronan-based formulas for not only tissue rejuvenation but also regeneration. Profhilo Structura^®^, or HL 90, studied here in comparison to HHA, is a new class-III medical device, with highly concentrated hybrid complexes made up of high- and low-molecular-weight hyaluronan. The high concentration was shown to affect rheology and chain entanglements, making the complex more rigid than injectables based on the linear hyaluronan commercialized so far. The formulation proved fully compatible with fat tissue obtained by well-known surgical procedures. Its ability to sustain hASCs’ viability and differentiation was highlighted, also showing that the relevant biomarker modulation is better prompted by these complexes than by HHA. The in vitro results reported here have demonstrated that adipogenic markers, such as PPAR-γ, adiponectin, and leptin, have been prompted towards stromal cells differentiation. This phenomenon can eventually be obtained by stimulating fat pad stem cell niches. However, to better assess the full potential of this formulation, further clinical studies should be conducted on the combination of HL 90 and fat, not only in tissue augmentation and remodeling for rejuvenation but also towards tissue engineering for regenerative medicine.

## Figures and Tables

**Table 1:** Dynamic moduli and complex viscosity values derived at 1 Hz and 37 °C. ANOVA test was used to compare the differences among samples; * *p* < 0.05 with respect to fat; ^#^ *p* < 0.05 with respect to Fat/HL 90 3:1.

Sample	G′ (Pa) at 1 Hz	G″ (Pa) at 1 Hz	η* (Pa·s) at 1 Hz
Pure HL 90	95 ± 10 *^#^	105 ± 11 *^#^	23 ± 2 *^#^
Fat/HL 90 1:3	5000 ± 1600	910 ± 320	810 ± 260
Fat/HL 90 1:1	6500 ± 2100	1200 ± 410	1000 ± 330
Fat/HL 90 3:1	8150 ± 2800	1300 ± 560	1300 ± 440
Pure fat	11,500 ± 5300	2000 ± 1300	18,000 ± 870

**Table 2:** Residual storage and loss moduli (% with respect to the control) at 5, 15, and 25 min of incubation with BTH 10 U/mL. All calculations were derived from measurements performed at 37 °C, 2% of strain, and 2.5 Hz of frequency.

Sample	Residual G′ (%)			Residual G″ (%)		
	5 min	15 min	25 min	5 min	15 min	25 min
HL 90	76.9 ± 10.9	51.3 ± 8.3	37.8 ± 4.7	86.5 ± 10.9	68.5 ± 10.8	55.2 ± 9.2
HHA	49.6 ± 2.70	11.3 ± 2.2	4.8 ± 0.7	68.3 ± 1.6	26.0 ± 3.3%	12.0 ± 2.3

**Table 3:** Primer sequences used for the qRT-PCR.

Gene	Forward Primer	Reverse Primer
GAPDH	5′-TGCACCACCAACTGCTTAGC-3′	5′-GGCATGGACTGTGGTCATGAG-3′
PPAR-γ	5′-TCGAGGACAGCGAGGCC-3′	5′-TCGAGGGTGTAGCGTGTAGAG-3′
ADP	5′-CCGCTTACATGTATCACTC-3′	5′-ATACTGGTCGTAGGTGAAGA-3′
LEPTIN	5-CCATCCTGGGAAGGAAAATG-3′	5-CCCTTAACGTAGTCCTTGCAG-3′

## Data Availability

The raw data from which the figures and tables presented in this study were derived are available on request from the corresponding author.

## Supplementary Materials

The following supporting information can be downloaded at https://www.mdpi.com/article/10.3390/ijms25042019/s1.
