# Supplementary figures and images for: Highly Concentrated Stabilized Hybrid Complexes of Hyaluronic Acid: Rheological and Biological Assessment of Compatibility with Adipose Tissue and Derived Stromal Cells towards Regenerative Medicine
Source: Int J Mol Sci. 2024 Feb 7;25(4):2019. doi: 10.3390/ijms25042019 (PMC10888561; doi:10.3390/ijms25042019)

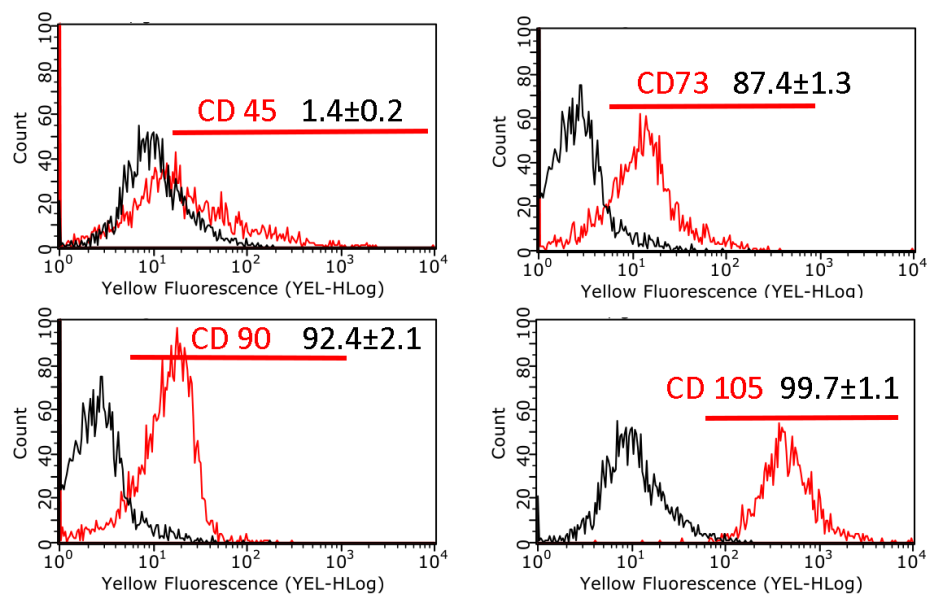

Figure S1

Supplement: Supplementary file 1 [file ijms-25-02019-s001.zip › ijms-2840526-supplementary.pdf]
